# In Memoriam: Cesar Noronha Raffin

**DOI:** 10.1055/s-0045-1814374

**Published:** 2025-12-22

**Authors:** Rubens José Gagliardi

**Affiliations:** 1 Faculty of Medical Sciences of Santa Casa de São Paulo, São Paulo SP, Brazil.

**Figure 1:**
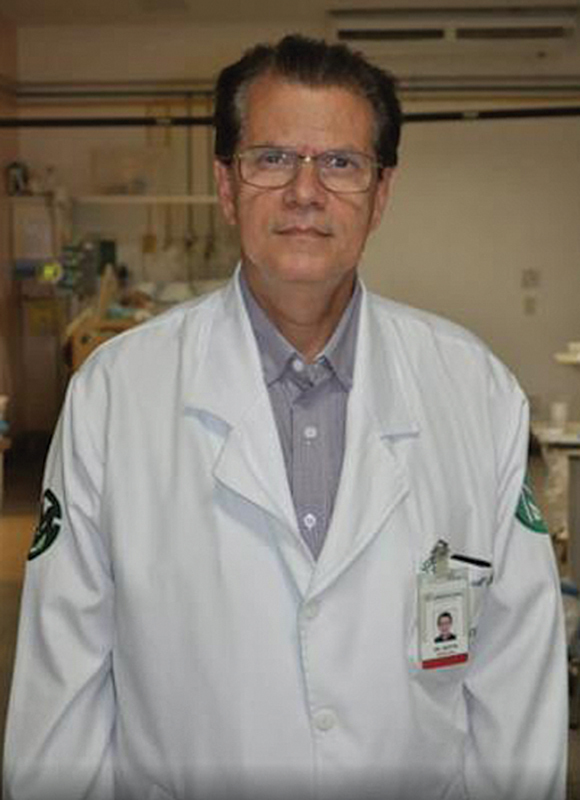
Professor Dr. Cesar Noronha Raffin.

With deep sorrow for the loss of our great colleague and friend Cesar, we present this brief tribute, recalling some details of his academic and professional life.

Born in the city of Porto Alegre, Brazil, he graduated in Medicine from Universidade Federal do Rio Grande do Sul in 1976. He completed his residency in Neurology at the then-called Department of Neuropsychiatry and Medical Psychology of Faculdade de Medicina de Ribeirão Preto, Universidade de São Paulo, from 1976 to 1979. From an early age, he expressed an interest and a vocation for academic life. He completed a Master's degree in Medicine (Neurology) at Universidade de São Paulo in 1981, under the guidance of Prof. Dr. Jorge Ambrust Figueiredo. He also completed his Ph.D. in Medicine (Neurology) at the same university in 1983, under the same advisor. He completed a postdoctoral fellowship at the University of Miami School of Medicine from 1986 to 1988 and another postdoctoral fellowship at the Beneficência Portuguesa de São Paulo from 2002 to 2004 in the field of Neurointervention. In 2001, he completed additional training at the International Medical School (MSc in Neurovascular III Mod.) at the Université Paris-Sud, France, and Mahidol University, Thailand.

After this brilliant training, he pursued an academic career, consistently achieving a high level of performance. He initially transferred to the city of Uberlândia, state of Minas Gerais, where he rose through the ranks at the School of Medicine of Universidade Federal de Uberlândia, reaching the rank of full professor. He remained at this university from 1984 to 1997. He worked at Universidade Federal de Goiás from 1997 to 1999 as an Assistant Professor of Neurology. He later transferred to the Neurology Department of Universidade Estadual Paulista “Júlio de Mesquita Filho” (UNESP), in the city of Botucatu, state of São Paulo, where he served as an Assistant Professor and headed the Department of Cerebrovascular Disease Research and Treatment, as well as the Department of Interventional Neuroradiology. He later transferred to the city of Vitória, state of Espírito Santo, where he worked as a Neurologist and Neurointerventionalist at the Hospital Meridional and its medical school. In these settings, he consistently demonstrated great enthusiasm for Neurology, particularly in the field of cerebrovascular diseases, with exemplary performance.

He was president of Sociedade Brasileira de Doenças Cerebrovasculares (the Brazilian Society of Cerebrovascular Diseases) from 2000 to 2004, demonstrating excellent leadership and organizational skills. He participated in the organization of the first guidelines in Neurology, specifically in the field of cerebrovascular diseases, as an author or coauthor in these publications, which had a significant impact on the field of Neurology. Notably, he coordinated the First Brazilian Consensus on the Use of Thrombolytics in Cerebrovascular Diseases in 2002. 1

He was a frequent speaker, with his name always remembered at various conferences, congresses, symposia, and meetings held in locations throughout Brazil and abroad. He always stood out for the quality of his presentations, as well as his excellent scientific and ethical standards and teaching methods.

He mentored several colleagues in their Master's and Ph.D. programs and served on numerous examination boards at the most renowned medical schools.

On March 14, 2022, he received an honor from the Regional Medical Council of Espírito Santo, in gratitude for his services.

He was a model professional and an inspiration to students and assistants. He trained several new colleagues in his field, who, following his teachings, expanded the excellent services and research on Neurology.

His primary vocation was research, teaching, and medical care, which he consistently carried out with mastery, integrity, and great kindness and optimism.

With a strong focus on cerebrovascular diseases and neurointervention, he developed several pioneering works, both nationally and internationally, and left numerous publications 2 3 4 5 6 that significantly contributed to the advancement of medicine, benefiting thousands of patients through his interventions.

It would never be too much to remember the person Cesar was: kind, ethical, straightforward, hardworking, always willing to research, teach, publish etc. He was a great physician, professor, friend, and a magnificent colleague.

Undoubtedly, his early passing leaves a void that is difficult to fill; yet he has left a great legacy through his example, contributions, and publications.
